# Medical Measures in Hypertensives Considered Resistant

**DOI:** 10.1093/ajh/hpad118

**Published:** 2023-12-20

**Authors:** Fadl Elmula M Fadl Elmula, Julian Eek Mariampillai, Sondre Heimark, Sverre E Kjeldsen, Michel Burnier

**Affiliations:** Division of Medicine, Ullevaal University Hospital, Cardiorenal Research Centre, Oslo, Norway; Heart Center, King Faisal Specialist Hospital and Research Center, Riyadh, KSA; Department of Medicine, Lovisenberg Diaconal Hospital, Oslo, Norway; Division of Medicine, Ullevaal University Hospital, Cardiorenal Research Centre, Oslo, Norway; Medical Faculty, Institute of Clinical Medicine, University of Oslo, Oslo, Norway; Department of Nephrology, Ullevaal University Hospital, Oslo, Norway; Division of Medicine, Ullevaal University Hospital, Cardiorenal Research Centre, Oslo, Norway; Medical Faculty, Institute of Clinical Medicine, University of Oslo, Oslo, Norway; Department of Cardiology, Ullevaal University Hospital, Oslo, Norway; Faculty of Biology and Medicine, University of Lausanne, Lausanne, Switzerland

**Keywords:** adherence, antihypertensive drugs, blood pressure, hypertension, refractory hypertension, treatment resistance

## Abstract

**BACKGROUND:**

Patients with resistant hypertension are the group of hypertensive patients with the highest cardiovascular risk.

**METHODS:**

All rules and guidelines for treatment of hypertension should be followed strictly to obtain blood pressure (BP) control in resistant hypertension. The mainstay of treatment of hypertension, also for resistant hypertension, is pharmacological treatment, which should be tailored to each patient’s specific phenotype. Therefore, it is pivotal to assess nonadherence to pharmacological treatment as this remains the most challenging problem to investigate and manage in the setting of resistant hypertension.

**RESULTS:**

Once adherence has been confirmed, patients must be thoroughly worked-up for secondary causes of hypertension. Until such possible specific causes have been clarified, the diagnosis is *apparent* treatment-resistant hypertension (TRH). Surprisingly few patients remain with *true* TRH when the various secondary causes and adherence problems have been detected and resolved. *Refractory* hypertension is a term used to characterize the treatment resistance in hypertensive patients using ≥5 antihypertensive drugs. All pressor mechanisms may then need blockage before their BPs are reasonably controlled.

**CONCLUSIONS:**

Patients with resistant hypertension need careful and sustained follow-up and review of their medications and dosages at each term since medication adherence is a very dynamic process.

## Graphical Abstract

**Graphical Abstract:**
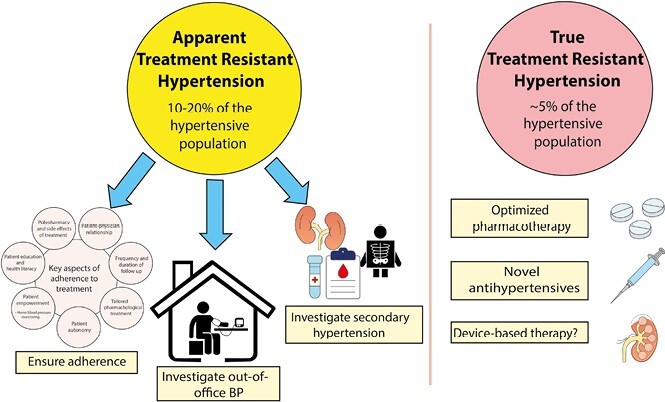

A hypertensive patient is considered resistant to treatment if he/she has uncontrolled blood pressure (BP) despite taking at least 3 antihypertensive drugs in maximally tolerated doses, one of which is a diuretic drug. Thus, in patients with treatment-resistant hypertension (TRH), ≥4 antihypertensive drugs are often prescribed to achieve BP control.^1^ According to another definition, a patient has *refractory* hypertension when BP remains uncontrolled on maximal or near-maximal therapy, which is the use of ≥5 antihypertensive agents of different classes, including a long-acting thiazide-like diuretic (such as chlorthalidone) and spironolactone.^2^ All general rules and guidance to treat patients with hypertension are mandatory and should be strictly applied in patients considered resistant to treatment, as this group of patients are those with the highest risk of developing complications of uncontrolled hypertension.^3^ The foundation of antihypertensive treatment is lifestyle interventions, consisting of, but not restricted to, low sodium and high potassium intake, modest alcohol consumption, weight loss if obesity is present, smoking cessation and physical exercise. In patients with resistant hypertension, these measures are, if possible, even more important than in mild and moderate hypertension because patients may then respond better to antihypertensive drugs.^4^ This review will focus on the pharmacological treatment and management of patients who are considered resistant or apparent resistant to treatment.

## PHARMACOLOGICAL THERAPY

Pharmacological therapy is the mainstay in the management of patients with uncontrolled BP who are at high risk of cardiovascular disease. These patients require close follow-up over time, with out-of-office BP measurements as an adjunct in BP estimation, and often a team-based and multidisciplinary approach. When initiating a new or adjusted drug regimen for hypertension, monthly follow-ups with evaluation of the response to treatment should be performed until recommended BP control is achieved,^5^ with the aim of BP control within the first 3 months.^6^ The use of out-of-office BP measurements, also during up-titration of medication, cannot be emphasized enough. Many patients with uncontrolled office BP have some degree of white coat hypertension, and wrongfully increasing medication may in itself lead to reduced adherence, as discussed later in this review. The use of home BP measurements has several advantages, including the importance of patient participation in the follow-up of hypertension, hopefully leading to increased adherence to the treatment.

### Selection of antihypertensive agents in resistant hypertension

**Figure 1.**
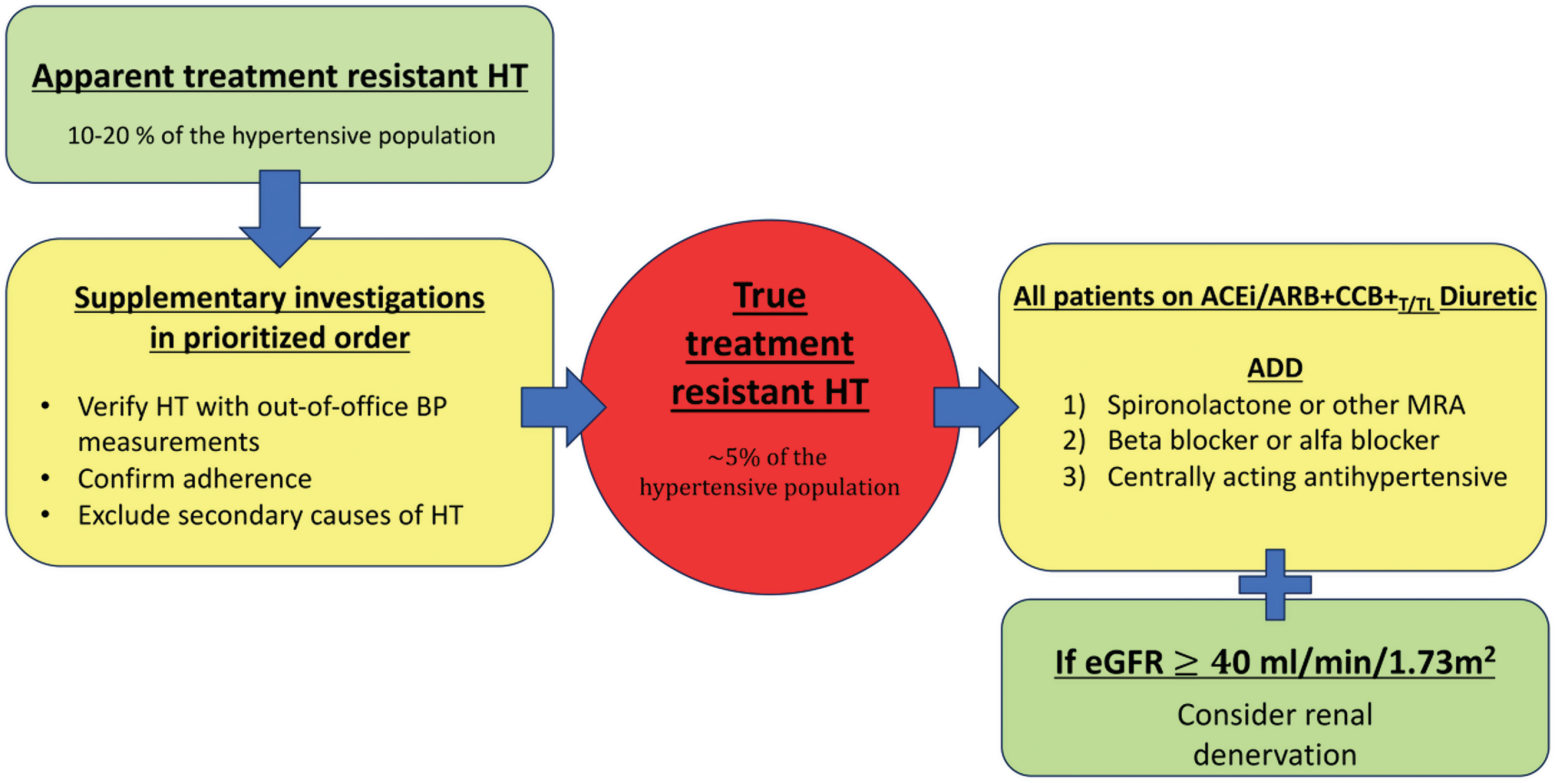
Assessment of apparent and true resistant hypertension. Abbreviations: ACEi, angiotensin-converting enzyme inhibitor; ARB, angiotensin receptor blocker; BP, blood pressure; CCB, calcium channel blocker; eGFR, estimated glomerular filtration rate; HT, hypertension; T/TL, thiazide/thiazide-like.

According to American College of Cardiology (ACC)/American Heart Association (AHA) 2017, International Society of Hypertension (ISH) 2020 and European Society of Hypertension (ESH) 2023 Guidelines, the preferred antihypertensive drug treatment of resistant hypertension should at least include the following antihypertensive classes: angiotensin-converting enzyme inhibitor/angiotensin receptor blocker, calcium antagonist (=calcium channel blocker), and thiazide diuretics.^5–7^ The steps recommended to apply if triple therapy (angiotensin-converting enzyme inhibitor/angiotensin receptor blocker, calcium channel blocker, and diuretic) fails to achieve optimal BP are the following (Figure 1):

- To optimize and tailor the current treatment regimen based on individual risk factors including health behavior changes.
- Use of thiazide-like rather than thiazide diuretics, and initiation of loop diuretics for estimated glomerular filtration rate <30 ml/min/1.73 m^2^ or clinical volume overload.^8^
- Add a low dose of spironolactone as the 4th line agent in patients whose serum potassium is <4.5 mmol/l and whose estimated glomerular filtration rate is >45 ml/min/1.73m^2^ to achieve BP targets.^9,10^
- If spironolactone is contraindicated or not tolerated, amiloride, beta-blocker, clonidine, doxazosin, and eplerenone are alternatives, or any available antihypertensive class not already in use.^1,11–13^

### Optimized pharmacotherapy

**Figure 2.**
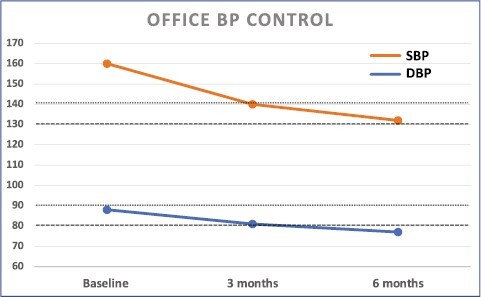
Office blood pressure (BP) response after adjusted therapy. Systolic (SBP) and diastolic (DBP) office BP (mm Hg) response to adjusted pharmacological therapy after 3 and 6 months in the Oslo RDN Study. Dotted lines representing treatment targets: small dots indicate treatment target at the time of the study (<140/90 mm Hg); large dots indicate present treatment targets (<130/80 mm Hg). Abbreviation: RDN, renal denervation.

**Figure 3.**
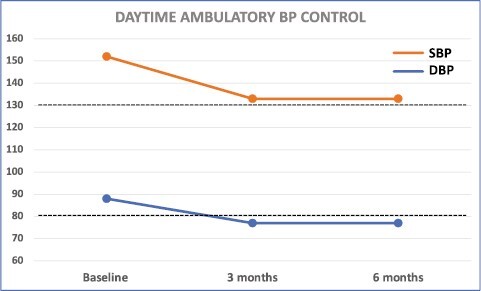
Daytime ambulatory blood pressure (BP) response after adjusted therapy. Systolic (SBP) and diastolic (DBP) daytime ambulatory BP (mm Hg) response to adjusted pharmacological therapy after 3 and 6 months in the Oslo RDN Study. Dotted lines (130/80 mm Hg) corresponding to treatment target for office BP at time of the study (<140/90 mm Hg). Abbreviation: RDN, renal denervation.

**Figure 4.**
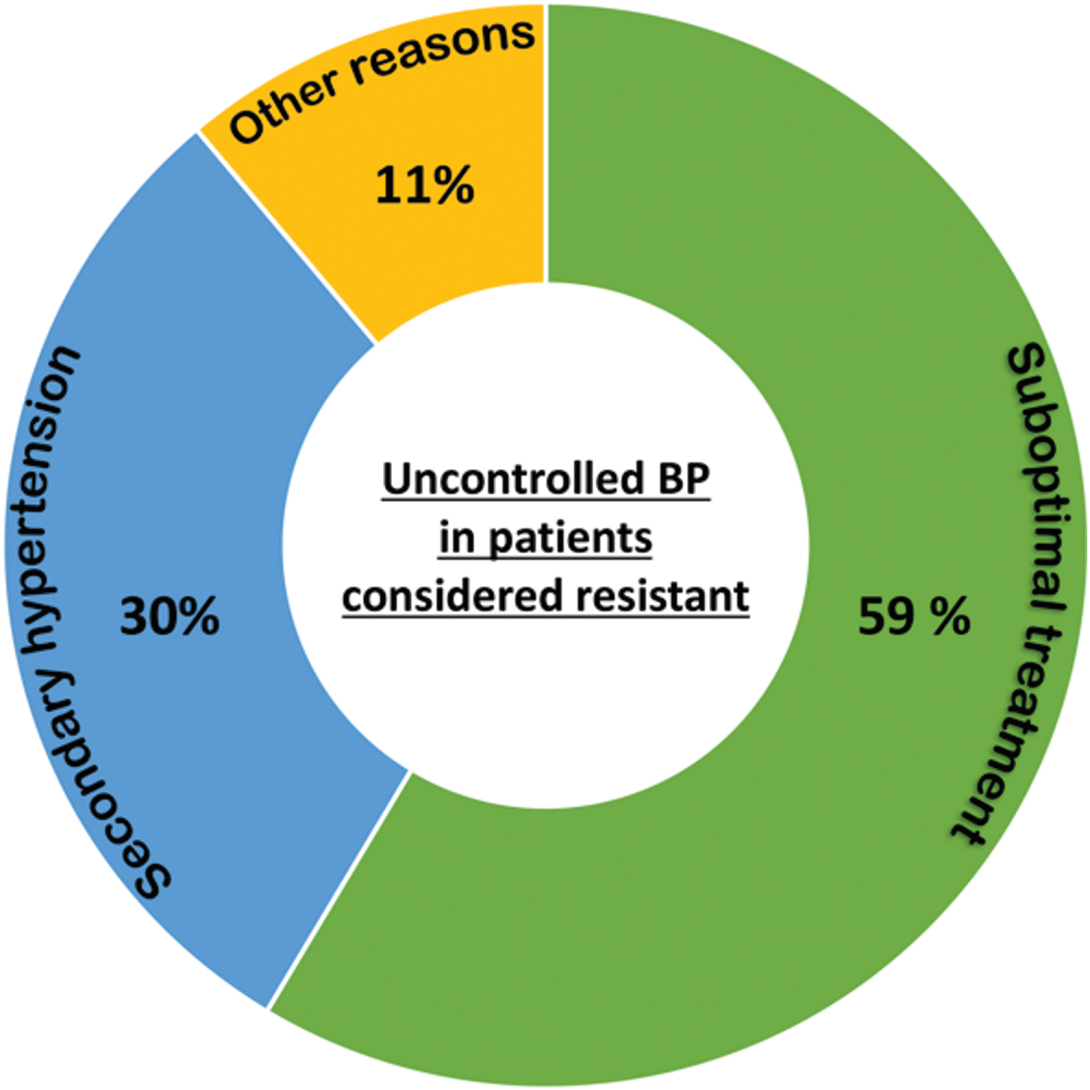
Uncontrolled blood pressure (BP) in patients considered resistant. Reasons for uncontrolled office BP in patients considered resistant to antihypertensive treatment in a tertiary assessment. Results based on exclusion from the Oslo RDN Study. Suboptimal treatment includes poor drug adherence, white coat hypertension, and drug adjustments. Other reasons include chronic autoimmune disease, language barrier, unstable angina pectoris, and excessive intake of alcohol or caffeine. Abbreviation: RDN, renal denervation.

The multifactorial etiology of hypertension should be reflected in the approach to pharmacotherapy of the treatment-resistant hypertensive patient. Both individual hemodynamic parameters and comorbidities should be considered when choosing the correct multidrug regimen.^14^ In addition to stepwise titration of antihypertensive drugs as per guidelines, bioimpedance cardiography-guided pharmacotherapy may help decision-making by indicating hemodynamic imbalances such as volume overload or high systemic vascular resistance especially in patients with difficult-to-control hypertension.^15^ In the Oslo RDN study, patients with true TRH were randomized to renal denervation (RDN) or adjustments of their drug regimen based on hemodynamic measurements.^16^ Adjustments were based on European hypertension guidelines in addition to data obtained using impedance cardiography. The drug-adjustment group had superior BP control after 6 months compared with RDN, and almost reached treatment target already at 3 months (Figures 2 and 3). Hence, BP control may be achievable also in patients considered to have true resistant hypertension, as demonstrated during the evaluation of the early era of RDN.^16,17^ This is often due to suboptimal assessment and initial treatment (Figure 4),^18^ and emphasizes the importance of distinguishing between apparent or true TRH. Although bioimpedance-guided pharmacotherapy was used in the Oslo RDN study in which it was superior to RDN, its use has been investigated in a randomized controlled trial without showing improved BP control compared with unguided optimalization of pharmacotherapy.^19^ The inability to use impedance cardiography should therefore not be considered as an excuse for not achieving treatment target. Of utmost importance is the initial close follow-up and state-of-the-art clinical assessments until target BP is achieved. This is particularly important in patients with severe or symptomatic hypertension, hypertension-mediated organ damage or intolerance to treatment. Some guidelines recommend as frequent as monthly or every 2 months initially, thereafter every 3 or 6 months when target BP is achieved.^20^ But even with the best tailor-made pharmacotherapy and close follow-up, BP control will not be achieved without proper adherence.

### Options when standard pharmacotherapy fails

Besides ensuring proper lifestyle interventions, confirming adherence and ruling out secondary causes of hypertension, the availability of new drugs specifically investigated in patients with resistant hypertension might be helpful in the future. Thus, in later years, novel drugs with antihypertensive effects have been developed. Both lorundrostat and baxdrostat, aldosterone synthase inhibitors, have shown clinically significant BP lowering effects. Lorundrostat and baxdrostat have been investigated as add-on to at least 2 or 3 other antihypertensives, respectively, in patients with resistant hypertension and show a dose-dependent BP reduction.^21,22^ As with mineralocorticoid receptor antagonists, hyperkalemia may occur. This can now easily be counteracted by peroral potassium binders. Non-steroidal mineralocorticoid receptor antagonists have recently been investigated in patients with type 2 diabetes and chronic kidney disease with both renal^23^ and cardiovascular endpoints^24^ with beneficial effects. These drugs do not have the same side effects as the regular mineralocorticoid receptor antagonists, and are also good antihypertensive alternatives.

The endothelin pathway as a contributor to hypertension pathophysiology has been known for a long time, but endothelin receptor antagonists have not reached a prominent position in antihypertensive treatment. With newer dual endothelin antagonists, these agents may prove useful as add-on medication in resistant hypertension.^25^

The latest possible adjunct presented is the quarterly or biannual subcutaneous injection of zilebesiran, an agent reducing hepatic angiotensinogen production, which has quite potent BP lowering effects without major adverse effects.^26^ Further research is needed before implementation of zilebesiran in routine clinical use. It is assumed that injections of long-acting agents will to a great extent ensure adequate adherence. It is also important to investigate the feasibility of reversal agents or the effect of different compensatory mechanisms as in cases where inhibition of the renin–angiotensin system is unfortunate, e.g., during intercurrent illness with hypotension and circulatory shock.

Also worth remembering is that all “cornerstone” drugs used in heart failure will also reduce BP. Hypertension is one of the leading causes of heart failure, and at least if there is any sign of heart failure or cardiac remodeling, these drugs may be considered in patients with resistant hypertension, if not already part of the drug regimen.^27^ This should be considered regardless of left ventricular ejection fraction, keeping in mind that treatment target may differ in presence of left ventricular hypertrophy.^28^

There may be several reasons for not being able to fully up-titrate medication, e.g., intolerable side effects, or merely a patient’s sincere desire to avoid taking pills daily. For these patients, device-based antihypertensive treatment may be an alternative. Currently, RDN is the only feasible option, and is also included in the latest ESH guidelines as an option for uncontrolled or resistant hypertension, although it is emphasized that it should only be performed in experienced specialized centers.^6^ The story of RDN has changed from optimism after the first proof-of-concept studies, to pessimism after the first sham-controlled study,^29^ and again to optimism with several later studies. It is now proven that RDN is safe and provides long-term BP reduction, and recently, 2 RDN devices have received FDA approval for clinical use. RDN seems to lower BP on average equally to 1 antihypertensive drug.^30^ Still, long-term BP reduction data are at best uncertain.^31^ Long-term data from the rather small Oslo RDN study indicate no difference in BP reduction between RDN and optimized pharmacotherapy alone, and these results provide the longest follow-up reported after RDN.^32^ Furthermore, there are a large portion of nonresponders to the procedure; between 20% and 30% of patients will not experience clinically significant BP reduction after the procedure. This leaves RDN as a final hope for only a small fraction of hypertensive patients, and not a first-line option intended for large-scale use. Even more important is that no study has yet shown benefit of RDN on hypertensive end-organ damage or hard cardiovascular endpoints. Although being a safe^33^ and, in short-term, effective BP lowering treatment option, the uncertainties regarding durability, difficult predictability, high cost compared with long-term gain and no evidence on hard cardiovascular endpoints, prevent RDN from being the obvious solution in true resistant hypertension. At last, one has to emphasize that in patients with resistant hypertension treated by RDN, the number of oral antihypertensive drugs is only modestly reduced and remains high, since the effect of RDN corresponds more or less to the effect of 1 antihypertensive drug. Hence, the promise of an improved adherence is often not kept.

## ADHERENCE AND PERSISTENCE TO ANTIHYPERTENSIVE THERAPY

**Figure 5.**
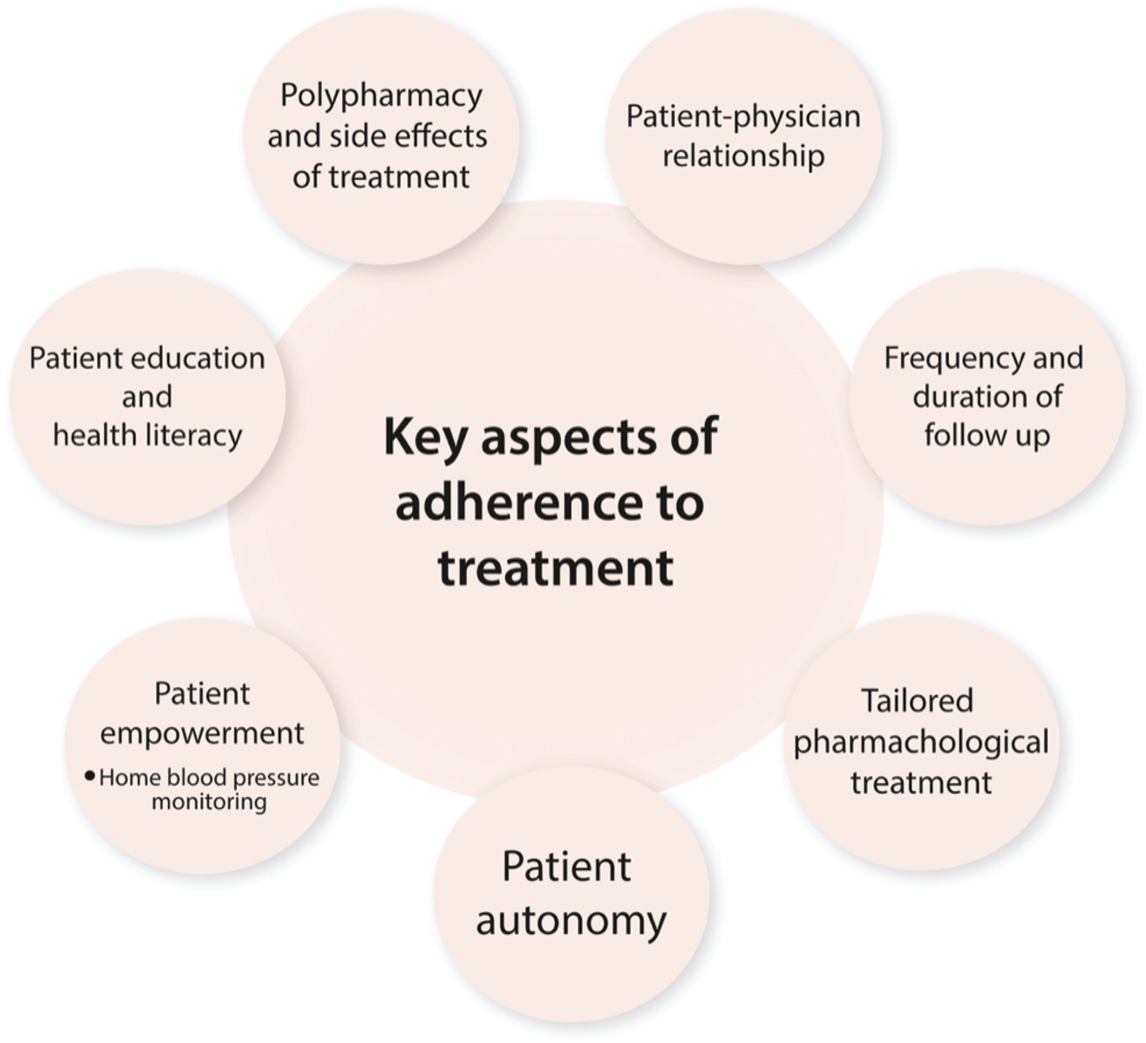
Aspects associated with reduced adherence. Different aspects that may be included in evaluation of complete or partial nonadherence to antihypertensive treatment.

Adherence to medical therapy is the ability of the patient to comply with the prescribed treatment while persistence to medical therapy refers to the degree of adherent behavior over time.^34^ Nonadherence and poor persistence to antihypertensive therapy are probably the main reasons for the high rates of uncontrolled BP and it is estimated to cause a substantial economic burden across several medical fields.^35–37^ Several studies, both retrospective and prospective, have indicated that drug nonadherence in hypertension is more common than previously thought, ranging up to 86% in apparent treatment-resistant patients (Table 1).^47,48^ This variation largely depends on the patient population and the method used to evaluate adherence.^38,43,49,50^ Causes of medication nonadherence are multifactorial and understanding the complex nature of its associated factors remains a topic in need of further investigation (Figure 5). As nonadherence has become an emerging topic of interest reaching beyond the field of hypertension, the World Health Organization has addressed 5 main factors associated with nonadherence—disease-related factors, therapy-related factors, socioeconomic factors, factors associated with the healthcare team and system in place, and patient-related factors.^51^ The 3 main factors specifically associated with nonadherence to antihypertensive therapy are patient-, physician-, and treatment-related factors.^52^

**Table 1.** Percentage of patients classified as nonadherent in different studies of apparent treatment-resistant hypertension

First author and reference no.	No. of patients	Method used	Total percentages classified as nonadherent^a^	Population and characteristics
Ceral^38^	84	TDM, blood sample	65.5%	Population: Patients with difficult-to-control hypertension. Inclusion: BP >150/95 mm Hg and a minimum of 3 antihypertensives from different classes.
Jung^39^	76	TDM, urine sample	53.0%	Population: Patients with uncontrolled hypertension. Inclusion: BP >140/90 mm Hg and a minimum of 3 antihypertensives from different classes.
Strauch^40^	163	TDM, blood sample	47.0%	Population: Outpatients with resistant essential hypertension. Inclusion: BP >140/90 mm Hg and a minimum of 3 antihypertensives including a diuretic.
Strauch^40^	176	TDM, blood sample	19.0%	Population: Hospitalized patients with resistant essential hypertension. Inclusion: BP >140/90 mm Hg and a minimum of 3 antihypertensives including a diuretic.
Fadl Elmula^41,42^	83	DOT + 24 h ABPM	29.3%	Population: Patients with apparent treatment-resistant hypertension evaluated for possible renal denervation. Inclusion: SBP >140 mm Hg and daytime SABPM >135 mm Hg, and a minimum of 3 antihypertensives including a diuretic.
Brinker^43^	56	TDM, blood sample	54.0%	Population: Patients with treatment-resistant hypertension. Inclusion: BP >140/90 mm Hg and a minimum of 3 antihypertensives including a diuretic.
Tomaszewski^44^	208	TDM, urine sample	25.0%	Population: New referrals, follow-up patients and referrals to possible renal denervation with hypertension. Inclusion: BP >140/90 mm Hg.
Florczak^45^	36	TDM, blood sample	86.1%	Population: Patients with primary resistant hypertension. Inclusion: Systolic BP >140 mm Hg, a minimum of 4 antihypertensives, and at least 1 sign of nonadherence^b^.
Hameed^46^	50	DOT + 24 h ABPM	50.0%	Population: Patients with uncontrolled hypertension evaluated for nonadherence. Inclusion: Reduction of SABP >5 mm Hg using DOT and 24 h ABPM, compared with previous non-DOT 24 h ABPM, and minimum 3 antihypertensives.

Abbreviations: ABPM, ambulatory blood pressure measurement; BP, blood pressure; DOT, directly observed therapy; SABP, systolic ambulatory blood pressure; SABPM, systolic ambulatory blood pressure measurement; SBP, systolic blood pressure; TDM, therapeutic drug monitoring.

^a^Total percentage classified as nonadherent include both partial and complete nonadherence.

^b^Signs of nonadherence include e.g., tachycardia while using an adequate dose of β-blocker or low potassium plasma levels when taking spironolactone.

### Patient-related factors

Patient-related factors contributing to medication nonadherence are many and diverse. Some factors, such as younger age, have a well-known association with nonadherence, while others, such as female gender, have been found only in some studies and remain disputed.^53–55^ Furthermore, health literacy remains an important factor in nonadherence, reflecting to some degree the overall understanding of a patients’ comprehension of their own disease, and the implications of not receiving adequate treatment.^56^ Even though several studies have explored the relationship between health literacy and nonadherence, existing studies on health literacy suffer from imprecise methods in detection of nonadherence, relying mainly on self-reports rather than objective methods.^57,58^ In addition to sociodemographic factors, certain psychological and behavioral factors may influence the degree of adherence, exemplified by the fact that nonadherence could be both intentional (i.e., the active choice of not taking medication as prescribed) and unintentional (i.e., forgetfulness).^59^ Particularly patients’ trust in medical treatment, and their beliefs about medicines have been extensively investigated, and play an important role in the persistence of adherent behavior.^60^ Bridging the gap between patient- and physician-related factors, joint decision-making and patient empowerment throughout the treatment process have been shown beneficial in improving adherence, yet many studies suffer from the same methodological limitations already mentioned.^61–64^

### Physician-related factors [physician inertia]

Physician-related factors contributing to nonadherence to antihypertensive drugs overlap somewhat with both patient- and treatment-related factors. The physician must be aware that prescription of complex treatment regimens, lack of joint decision-making or patient empowerment along with suboptimal communication between physicians with different areas of expertise or at different levels of care may contribute to decreased adherence.^64,65^ Therapeutic inertia is a very frequent and underestimated issue that might contribute to apparent treatment failures.^66–68^ As physician inertia might lead to a higher rate of treatment failure, one might speculate that this will further aggravate medication adherence, as patients who do not reach their treatment goals might be more likely to discontinue both medical treatment and lifestyle changes.^69,70^ Inertia may be extended to up-titrating study medications in randomized clinical trials (investigator inertia), an important issue previously addressed.^71^ Thus, apparent TRH in some of the world’s most cited randomized controlled trials in hypertension research was caused by investigator inertia.

### Treatment-related factors

Treatment-related factors associated specifically with low adherence to antihypertensive drugs include a higher number of prescribed daily pills, a higher number of prescribed antihypertensive pills, along with a higher number of prescribed concomitant agents.^72,73^ Such findings reflect existing physician concerns; that complex multipill treatment strategies are difficult to adhere to and maintain.^74^ This is reflected in the latest ESH hypertension guidelines, where rapid BP control and early introduction of combination-agent pills are emphasized to counteract physician inertia and complex medication regimens.^6,75^ A recent review and meta-analysis have shown that the use of single-pill combinations is associated with an improved BP control but also an improved adherence and persistence.^74^ Moreover, data from a German survey have confirmed that antihypertensive combination therapy reduces all-cause mortality and cardiovascular events when provided as a single pill compared with identical drugs as multipills.^76^

## ASSESSMENT OF NONADHERENCE

Both direct and indirect methods of assessing nonadherence exist (Table 2), all with advantages and disadvantages.^47,75^ Indirect methods of assessing drug nonadherence are readily applicable and cost-efficient in their use. However, since these methods make use of a surrogate for evaluation (such as BP in response to treatment), they do not prove actual intake of medication but rather the effect of taking the drug.^77^ Perhaps the most applied method is patient questionnaires, while a qualified guess made by a healthcare professional yielded no more accurate results than random chance.^78^ The use of questionnaires is a common method for collecting data on adherence, with more than 40 different types available.^79^ By far the most used questionnaire is the Morisky 8-item adherence scale, but even this underestimates adherence when verified against direct methods.^80^ Moreover, recent concerns have been raised regarding the validity of this questionnaire. This has led to the retraction of several papers by the authors of the questionnaire. According to Ortiz who demonstrated the flaws of this questionnaire “the MMAS-8 scores may be no more accurate in detecting patients with uncontrolled [blood pressure], than tossing a coin to decide.”^81^ Thus, today, this questionnaire should be avoided. Pill counting and cross-checking with prescription refill registries are also commonly applied but remain imprecise.^82–84^ An indirect method that has proven to be accurate when verified against direct methods includes electronic monitoring. This technology has been available for some time, and usually consists of a device that registers the physical maneuvers needed to remove a drug from its packaging, allowing a detailed record of dispensing events.^85^ This method is of course, based on the presumption that removing a dose from a pillbox predicts actual intake of the drug. Yet, the most useful information comes from the non-openings of the pillbox which are clearly associated with nonadherence. Today, this approach is used essentially in phase 2 and 3 clinical trials as it is supported by the Food and Drug Administration.^86^

**Table 2.** Methods to investigate drug adherence in hypertension

Indirect methods		Direct methods	
Patient self-report	Medication monitoring	Verification of intake	Chemical adherence testing
Questionnaires	Manual pill counting	Direct observed treatment	Blood sample analysis
Patient diary	Electronic pill box	Tele-monitoring	Urinalysis
Interviews	Prescription registries		Oral fluid analysis

Although less cost-effective and more labor-intensive than indirect methods, direct methods of assessing adherence to antihypertensive therapy are far more sensitive in that they prove actual intake of medication.^87^ In addition, it is shown that there is no overlapping between direct and indirect methods.^50,75^ Even though these methods are more accurate, they could be perceived as intrusive by patients. A commonly used method is witnessed intake of drugs or “directly observed treatment” that guarantees same-day adherence, but does not give any insight into the persistence of this behavior.^88^ Tele-monitoring [or “digital medicines”] is based on the ability to detect a small electronic device incorporated inside the drug in question that is activated when the drug is dissolved in the digestive system and detected by a noninvasive device.^89^ Though not routinely used in the context of treating hypertension, such telemedicine has been used in certain tuberculosis- and psychiatric treatment regimens.^90^

Lastly, and perhaps most important, is the use of chemical adherence testing (CAT).^91^ CAT is based on detection of drugs or drug metabolites in urine or serum. Urine drug or drug metabolite detection is at present limited to quantifying whether the drug is present or not and some drugs, for example telmisartan, does not have renal elimination. Furthermore, some drugs may be present for days after the last dose. On the other hand, measurements of serum drug concentrations may be used not only to detect antihypertensive drugs, but also allows quantification of drug levels in serum.^92^ As a measure toward practical use for clinicians, serum reference ranges 12–24 hours after the last dose for the most commonly prescribed (in Norway) antihypertensive drugs, measured by ultra-high-performance liquid chromatography coupled with tandem mass spectrometry, has been suggested.^44^ We believe these values may be useful for clinicians or departments seeking to utilize CAT in management of difficult-to-control hypertensives and have replicated the serum reference ranges in Table 3. The serum reference ranges were calculated based on pharmacokinetic properties and were validated against a limited sample of patient measurements. It should be kept in mind that these ranges are not correlated to drug response, i.e., BP lowering effect. A possible advantage of this method is that it can be used to optimize drug dosage and prescription, providing a potential for personalized medicine. In addition, it provides information regarding the biological half-life of the drugs.^48^ Despite its advantages, these methods are confounded by variations in pharmacokinetics and pharmacodynamics, of which ultrarapid metabolism because of gene polymorphism amplifications of CYP2D6 (rare) and the use of enzyme inducing drugs are the most relevant regarding non-detection despite adherent patient. Metabolic pathways of antihypertensive drugs with available serum reference ranges are presented in Table 3. Furthermore, serum measurements are not suitable for some antihypertensive drugs with short half-lives, such as loop diuretics and certain antihypertensives recommended for use in hypertensive emergencies and urgencies. It also provides only a momentary reflection of drug adherence and hence can be strongly influenced by the so-called white coat adherence, according to which, adherence improves before and after medical consultations. Moreover, it provides little information about persistence to drugs unless measurements are repeated over time. However, with these shortcomings in mind, CAT remains a promising and maybe the most reliable and easy to implement method for assessing adherence in antihypertensive treatment.

**Table 3.** Suggested serum reference ranges 12–24 hours after last dose, regardless of daily dosing frequency

Antihypertensive drug	Metabolic elimination (minor pathways in brackets)	Suggested serum concentration 12–24 hours after last dose, nmol/l
Alpha blockers		
Doxasozin	CYP 3A4 (2D6, 2C9)	5–80
Beta blockers		
Atenolol	None	75–750
Bisoprolol	CYP 2D6 (3A4)	10–200
Carvedilol	CYP 2D6, 2C9 (3A4, 2C19, 1A2, 2E1)	2.5–50
Labetalol	Conjugation	50–1,000
Metoprolol	CYP 2D6	10–500
Propranolol	CYP 2D6 (1A2)	50–400
Calcium channel blockers		
Amlodipine	CYP 3A4/5	10–40
Diltiazem	CYP 3A4 (2D6)	100–500
Lercanidipine	CYP 3A4	0.2–5
Nifedipine	CYP 3A4	20–150
Verapamil	CYP 3A4 (1A2, 2C8, 2C19, 2C18)	40–400
Angiotensin-converting enzyme inhibitor		
Enalaprilat	Carboxylesterase	10–300
Lisinopril	None	10–300
Ramiprilat	Carboxylesterase	4–60
Angiotensin receptor blockers		
Candesartan	CYP 2C9 (minor pathway)	15–200
Irbesartan	Conjugation (2C9 10%)	300–3,000
Losartan carboxylic acid	CYP (2C9, 3A4 14%	30–350
Telmisartan	Conjugation	8–80
Valsartan	CYP 2C9 (minor pathway)	3,000–4,000
Thiazide diuretics		
Bendroflumethiazide	70% (unknown enzymes)	1.5–30
Hydrochlorothiazide	None	15–300
Potassium sparing diuretics		
Canrenone	Not reported	15–300
Eplenerone	CYP 3A4	3.5–350

Table 3 reproduced from Rognstad *et al*.^92^ with permission.

In patients with uncontrolled hypertension despite the prescription of several antihypertensive medications, we need to determine if true TRH is present or merely apparent TRH. As the latter comprises a wide spectrum of causes for uncontrolled BP, these patients require thorough assessment from the alert clinician. Furthermore, the pharmacological therapy must be tailored to each patient, in order to maximize adherence to treatment and minimize adverse effects; this approach may be effective to achieve target BP always remembering that “one size does not fit all” (Table 4). The development of newer agents may also expand the antihypertensive arsenal, and medication used in other conditions, such as heart failure or chronic kidney disease, may have useful BP lowering effects. Nevertheless, drug adherence remains the most challenging problem to investigate and to manage in patients with TRH. Medication adherence issues are so common in resistant hypertension^48^ that they should be investigated before performing any costly investigations looking for secondary causes of hypertension. However, the topic of adherence and persistence clearly warrants further research including high-quality prospective interventional trials using interventions that have been studied and found to be effective at improving patient adherence.^93^

**Table 4.** Key steps to improve adherence

Key steps
Close initial follow-up
Ensure proper patient education and empowerment
Assess periodically the potential barriers to drug adherence
Simplify treatment regimen
Prefer long-acting antihypertensive drugs
Investigate side effects of treatment and adjust therapy accordingly
Ensure adequate long-term follow-up involving other healthcare professionals if needed
Monitor adherence in high-risk patients whenever possible

## Data Availability

Not relevant for this review article.

## Notes

All author takes responsibility for all aspects of the reliability and freedom from bias of the data presented and their discussed interpretation.
