# Correction to: Medical Measures in Hypertensives Considered Resistant

**DOI:** 10.1093/ajh/hpae028

**Published:** 2024-03-16

This is a correction to: Fadl Elmula M Fadl Elmula, Julian Eek Mariampillai, Sondre Heimark, Sverre E Kjeldsen, Michel Burnier, Medical Measures in Hypertensives Considered Resistant, *American Journal of Hypertension*, 2023; hpad118, https://doi.org/10.1093/ajh/hpad118

In the originally published version of this manuscript, the source of Table 3 was omitted. The article has been corrected to indicate that Table 3 reproduced from the following paper with permission:

Rognstad S, Soraas CL, Bergland OU, Hoieggen A, Strommen M, Helland A, Opdal MS. Establishing serum reference ranges for antihypertensive drugs. *Ther Drug Monit* 2021; 43:116–125.

